# Carpal spasm in a girl as initial presentation of celiac disease: a case report

**DOI:** 10.1186/s13256-017-1376-2

**Published:** 2017-09-04

**Authors:** Atifete Ramosaj-Morina, A. Keka-Sylaj, V. Hasbahta, A. Baloku-Zejnullahu, M. Azemi, R. Zunec

**Affiliations:** 1 Clinic of Pediatry, University Clinical Center of Kosovo, Pristina, Kosovo; 2 Clinical Hospital Center Zagreb, Tissue Typing Center, Zagreb, Croatia

**Keywords:** Celiac disease, Carpal spasms, Hypocalcemia

## Abstract

**Background:**

Celiac disease is an immune-mediated disorder elicited by ingestion of gluten in genetically susceptible persons. This disorder is characterized by specific histological changes of the small intestine mucosa resulting in malabsorption. This case was written up as it was an unusual and dramatic presentation of celiac disease.

**Case presentation:**

We report the case of a 3-year-old Albanian girl who presented at our clinic with carpal spasms and hand paresthesia. A physical examination at admission revealed a relatively good general condition and body weight of 10.5 kg (10 percentile). Carpal spasms and paresthesias of her extremities were present. Neuromuscular irritability was demonstrated by positive Chvostek and Trousseau signs.

Blood tests showed severe hypocalcemia with a total serum calcium of 1.2 mmol/L (normal range 2.12 to 2.55 mmol/L), ionized calcium of 0.87 (normal range 1.11 to 1.30 mmol/L), and 24-hour urine calcium excretion of 9.16 mmol (normal range female <6.2 mmol/day).

Among other tests, screening for celiac disease was performed: antigliadin immunoglobulin A, anti-tissue transglutaminase, and anti-endomysial immunoglobulin A antibodies were positive. A duodenal biopsy revealed lymphocyte infiltration, crypt hyperplasia, and villous atrophy compatible with celiac disease grade IIIb according to the Marsh classification. Following the diagnosis of celiac disease, human leukocyte antigen typing was performed, giving a definite diagnosis of celiac disease. She was started on a gluten-free diet.

Due to failure to follow a gluten-free diet, episodes of carpal spasms appeared again. Unfortunately, at the age of 7 years she presents with delayed psychophysical development.

**Conclusions:**

Although hypocalcemia is a common finding in celiac disease, hypocalcemic carpal spasm is a rare initial manifestation of the disease. Therefore, the possibility of celiac disease should be considered in patients with repeated carpal spasms that seem unduly difficult to treat. This should be evaluated even in the absence of gastrointestinal symptoms since hypocalcemia and its manifestation may present as initial symptoms of celiac disease even in young children.

## Background

Celiac disease (CD) is an immune-mediated disorder elicited by the ingestion of gluten and related prolamins from rye and barley in genetically susceptible persons. This disorder is characterized by specific histological changes (not pathognomonic) of the small intestine mucosa (especially proximal part) and malabsorption with consequences for growth and development delays.

Due to malabsorption, it is characterized by a number of substance deficiencies such as iron, folic acid, hypoproteinemia, and hypocalcemia. Hypocalcemia in CD may arise from the impaired absorption of vitamin D or the loss of calcium (Ca) through the binding of intraluminal Ca to unabsorbed fatty acids. The hallmark of acute hypocalcemia is neuromuscular irritability, although paresthesias of the extremities may also occur, along with fatigue and anxiety. In addition, very painful muscle cramps may develop and can progress into carpal spasms or tetany [1].

Hypocalcemia as a feature of CD is well described. In a cross-sectional study of 176 patients with CD, vitamin D deficiency was found to be present in 44.5%, whereas only 5.7% had hypocalcemia [2]. In another case series of 42 patients with CD, symptoms of tetany were present in 10% [3]. This case was written up because it was an unusual and dramatic presentation of CD. In all patients with CD, the enteropathy and associated symptoms will reverse on a gluten-free diet (GFD).

## Case presentation

A 3-year-old Albanian girl, from a rural area, was admitted to the Department of Gastroenterology at the University Clinical Centre of Kosovo, due to carpal spasms and hand paresthesia; no other associated symptoms were reported. A physical examination at admission revealed a relatively good general condition; her weight was 10.5 kg (10 percentile). Carpal spasms and paresthesias of her extremities were present. Neuromuscular irritability was demonstrated by positive Chvostek and Trousseau signs.

Personal and disease history: she is the fourth child in the family, from a well-controlled full-term pregnancy, with no history of drugs use or exposure to radiation. Delivery was completed in the regional hospital; she had a birth weight (BW) of 4300 gr. Retinol & cholecalciferol (AD3) prevention and vaccination were irregular. She was breastfed for 1 year, while supplementary food was introduced at sixth month of age.

She had several hospitalizations in the regional hospital. The first one occurred when she was 1-year old due to generalized seizure in which carpal spasms predominated. The other hospital admissions were usually for the same signs, that is, carpal spasms; tetany resolved following intravenous administration of Ca gluconate, without performing further tests.

At the age of 3 years, due to prolonged spasms, she was referred to a pediatric clinic. Blood tests showed severe hypocalcemia, with a total serum Ca of 1.2 mmol/L, normal range (NR) 2.12 to 2.55 mmol/L, ionized Ca of 0.87 mmol/L (NR 1.11 to 1.30 mmol/L), 24-hour urine Ca excretion of 9.16 mmol (NR female <6.2 mmol/day), low serum potassium of 3.0 mmol/L (NR 3.5 to 5.5 mmol/L), magnesium of 0.40 mmol/L (NR 0.65 to 1.05 mmol/L), and phosphorus 0.57 mmol/L (NR 0.8 to 1.45 mmol/L). Other significant results included hemoglobin 10.1 g/dL (NR 13 to 18 g/dL), mean corpuscular volume 98.1 fl (NR 82 to 98 fl), serum iron of 15 μg/dL (25 to 170 μg/dL), albumin 32 g/L (NR 34 to 48 g/L), and alkaline phosphatase 320 IU/L (NR 47 to 141 IU/L). Her serum total bilirubin, glycemia, urea, and creatinine levels were normal. Subsequent results revealed vitamin D deficiency with a low serum 25-OH vitamin D of 20 nmol/l (NR 25 to 137 nmol/L) and a raised serum parathyroid hormone of 35.6 pmol/L (NR 1.2 to 5.8 pmol/L). The results of thyroid function tests were normal: thyroid stimulating hormone 0.87 mIU/L (NR 0.35 to 4.5 mIU/L), triiodothyronine (T3) 2.69 nmol/L (0.9 to 2.8 nmol/L), and thyroxin (T4) 12.66 pmol/L (10 to 23 pmol/L).

Among other tests, screening for CD was performed, resulting l positive: antigliadin immunoglobulin A of 72.2 U/mL (cut-off 12 U/mL), anti-tissue transglutaminase IgA level of 33.9 (cut-off 10 U/ml), and anti-endomysial IgA antibodies (anti endomysium antibodies (EMA); 1:64).

An abdominal ultrasound revealed increased peristalsis, a more relaxed bowel, which was filled with liquid, and edema of the mucosa in the distal part of her digestive tract. There were no pathological findings in her liver and spleen.

In order to examine the upper part of her gastrointestinal system, a gastroduodenoscopy was performed, which revealed hyperemia in her postbulbar duodenum. Multiple biopsies were obtained during upper endoscopy, from the bulb (two biopsies) and from the second/third portion of her duodenum (four biopsies). CD was histologically confirmed by duodenal biopsies, which revealed lymphocyte infiltration, crypt hyperplasia, and villous atrophy, compatible with CD grade IIIb according to the Marsh classification [4].

Subsequently, genetic tests were performed that showed DQ2 heterodimer positive in cis position which reinforced the diagnosis of CD: human leukocyte antigen (HLA) A*24,*29; B*08,*14; DRB1*01, *03; DQA1*01:01, 05:01; DQB1*02,*05.

Initially she was treated with intravenously administered infusions of Ca, potassium, and magnesium, followed by oral supplements of Ca, potassium, vitamin D, and multivitamins. Following the diagnosis of CD, she was placed on a GFD. She was discharged from our hospital after 2 weeks. During a follow-up visit, 2 months later, no further episodes of carpal spasms were reported; there were lower titers of CD-specific antibody followed by normalization after 8 months on GFD.

She did not return for further follow-up visits. Four years later she was admitted again to our hospital because of a 24-hour history of carpal spasm. Recently, progressive malaise, anorexia, diarrhea, abdominal pain, and weight loss were also present. Despite our recommendation she had not adhered to a GFD during the past years. A subsequent physical examination revealed a lean child; she weighed 17 kg (under fifth percentile). She was uncommunicative, anxious, and irritable. She had dry pale skin and her adipose tissue was diminished at all levels. She had a distended abdomen, poor nutritional status, and poor psychophysical development.

She came from a rural area. She lives with a big family on a low income, therefore, their poor socioeconomic status led to GFD cessation. As a result, unfortunately, at the age of 7 years, she presented with delayed psychophysical development.

## Discussion

CD is the only treatable autoimmune disease, provided that a correct diagnosis is achieved and a strict lifelong GFD is implemented. Endoscopy with biopsy as an invasive procedure is considered the diagnostic gold standard, but it has been recently questioned. It is considered clinically useful to shift to a quantitative approach that can be defined as the “4 out of 5” rule; the diagnosis of CD is confirmed if at least four of the following five criteria are satisfied: typical symptoms of CD; positivity of serum CD IgA class autoantibodies at high titer; HLA-DQ2 or DQ8 genotypes; celiac enteropathy at a small bowel biopsy; and response to a GFD [5].

At the beginning, our patient had no history of chronic diarrhea and weight loss as frequent symptoms of CD; however, recurrent carpal spasms indicated the necessity for further investigations. Since serological test, biopsy, and genetic test were positive, and there was also a good response to GFD, the criteria for confirming the diagnosis of CD were met.

The mechanism of hypocalcemia in CD is complex. Ca malabsorption is due to villous atrophy in the proximal intestine. Villous atrophy causes a reduction in active absorption of Ca and an increase in unbound intraluminal fatty acids that bind intraluminal Ca. Ca malabsorption causes secondary hyperparathyroidism, and the latter results in an enhanced metabolic breakdown of vitamin D metabolites [6–8].

Although hypocalcemia is a common finding in CD, occurring in 5 to 10% of cases at diagnosis, hypocalcemic carpopedal spasm is a rare initial manifestation of the disease. Only a few reports describe symptomatic hypocalcemia as presentation of CD in childhood [9]. On the other hand, osteomalacia in adult patients with CD is a well-known presentation [10–13].

## Conclusions

Although hypocalcemia is a common finding in CD, hypocalcemic carpal spasm is a rare initial manifestation of the disease. Therefore, the possibility of CD should be considered in patients with repeated carpal spasms that seem unduly difficult to treat. This should be evaluated even in the absence of gastrointestinal symptoms since hypocalcemia and its manifestation may present as initial symptoms of CD even in young children.

## Abbreviations

Ca: Calcium
CD: Celiac disease
GFD: Gluten-free diet
HLA: Human leukocyte antigen
NR: Normal range
